# Gesundheit – digital und inklusiv: eine Lernsoftware barrierearm gestalten

**DOI:** 10.1007/s11553-021-00896-z

**Published:** 2021-09-06

**Authors:** Lena Sube, Jessica Bröhl, Lisa Kadatz, Inga Klose, Stefanie Frings, Jana York

**Affiliations:** Fakultät Rehabilitationswissenschaften, Technische Universität Dortmund, Emil-Figge-Str. 50, 44227 Dortmund, Deutschland

**Keywords:** Arbeitsschutz, Lernschwierigkeiten, Digitale Teilhabe, Behindertenhilfe, Partizipative Forschung, Occupational safety, Learning disabilities, Digital participation, Social services, Accessibility

## Abstract

**Hintergrund:**

Der Kompetenzerwerb in den Bereichen Arbeitsschutz und Gesundheit ist für Menschen mit Lernschwierigkeiten auf Grund vielfältiger Barrieren erschwert. Die softwarebasierte Vermittlung von gesundheitsbezogenen Kompetenzen stellt eine Möglichkeit dar, die Barrieren in der Gesundheitsbefähigung abzubauen.

**Methodik:**

Es wurden drei leitfadengestützte Fokusgruppengespräche mit Beschäftigten mit Lernschwierigkeiten und Mitarbeitenden einer WfbM (Werkstatt für Menschen mit Behinderung) der Recklinghäuser Werkstätten GmbH geführt. Im Rahmen dessen wurden Kenntnisse zu Gesundheitsförderung und Arbeitsschutz sowie die bevorzugte Lernmethode erfragt. Außerdem wurden Gestaltungskriterien und Entwürfe für barrierefreie Lerninhalte diskutiert. Hierfür stand die Lernsoftware sam® von secova GmbH & Co. KG, Rheine, beispielhaft zur Verfügung. Die gewonnenen Erkenntnisse wurden in die Revision der Lerninhalte einbezogen und als Handreichung für die barrierefreie Gestaltung digitaler Lerninhalte für die Zielgruppe mit Lernschwierigkeiten aufbereitet.

**Ergebnisse:**

Anhand multimedialer Inhalte sollte eine didaktische Vielfalt angeboten werden. Zum besseren Verständnis sollten hierbei vereinfachte Sprache und Symbolbilder sowie eine kontrastreiche und übersichtliche Gestaltung verwendet werden. Der Fokus sollte auf den individuellen Bedürfnissen der Adressierten sowie den Gegebenheiten der Einrichtung liegen. Zum Abbau von Barrieren sollten verschiedene didaktische und multimediale Materialien zur Wissensvermittlung genutzt werden.

**Schlussfolgerung:**

Der Einsatz der Handreichung ist auch in anderen Kontexten vorstellbar und sollte in der Praxis evaluiert werden.

## Abstract (en)

**Background:**

Acquiring occupational health and saftety competencies is difficult for peole with learning difficulties due to a variety of barriers. Software-based teaching of health-related topics is a possibility to reduce barriers to health empowerment for people with learning disabilities.

**Materials and methods:**

This article aims to determine practical recommendations for action from these aspects. These were derived from a qualitative and participative research project that was carried out in the form of three focus groups. Participants were employees with and without disabilities of a workshop for handicapped people, the WfbM Recklinghäuser Werkstätten GmbH. Discussions with focus groups were conducted. The participants were shown accessible drafts for the learning software sam® (secova GmbH & Co. KG, Rheine, Germany) on the subject of occupational safety. Those drafts were then discussed and evaluated jointly. The knowledge gained was then included into the revision of the software design and was edited into a manual for practitioners.

**Results:**

The research found that fundamental aspects like a high-contrast and clear design, the use of simple language or the support of symbol icons need to be taken into account. Furthermore, the focus has to be both on the individual needs of people with disabilities and the local circumstances of the institutions.

**Conclusion:**

The use of the manual is also conceivable in other contexts. Prospectively the results should be evaluated in practice.

## Einleitung

Nach der Ottawa-Charta zielt Gesundheitsförderung „auf einen Prozess, allen Menschen ein höheres Maß an Selbstbestimmung über ihre Gesundheit zu ermöglichen und sie damit zur Stärkung ihrer Gesundheit zu befähigen“ [13]. Auch die Behindertenrechtskonvention der Vereinten Nationen (UN-BRK) fordert „ein Höchstmaß an Gesundheit“ [11]. So sollten ganzheitliche Maßnahmen zur Stärkung der physischen und psychischen Gesundheit für alle Menschen entwickelt werden. In der Arbeitswelt hat sich dafür das betriebliche Gesundheitsmanagement u. a. mit Maßnahmen zum Arbeitsschutz etabliert [10].

## Aktueller Forschungsstand

Trotz der rechtlichen Forderungen zeigt sich eine geringe Gesundheitsbefähigung von Menschen mit Lernschwierigkeiten^1^ [5, 14, 19]. Außerdem fehlt es an umfassenden Studien zur Gesundheitsversorgung von Menschen mit Beeinträchtigungen [4] sowie an adressat*innenbezogenen Konzepten. Es deutet sich an, dass Menschen mit Beeinträchtigungen in der Gesundheitsversorgung zahlreichen baulichen Barrieren ausgesetzt sind [3], bspw. durch fehlende barrierearme Zugänge und Blindenleitsysteme zu Arztpraxen. Außerdem begegnen diesen Personen im Alltag strukturelle Barrieren bspw. in Form von knapp bemessenen Personalschlüsseln in Einrichtungen der Behindertenhilfe, die nur eine begrenzte Betreuung bei Arztbesuchen, Ernährungskursen o. Ä. ermöglichen. Die Anzahl an Krankheitstagen von Menschen mit Beeinträchtigung fällt deutlich höher aus als jene von Menschen ohne Beeinträchtigung [4]. Zudem gelten diese Personen hinsichtlich der COVID-19-Pandemie („coronavirus disease 2019“) als Risikopatient*innen [15] und haben somit einen besonderen Bedarf an gesundheitlicher Aufklärung und barrierearmen Konzepten. In diesem Kontext stellen auch digitale Inhalte, wie sie aufgrund der COVID-19-Pandemie verstärkt Anwendung finden [7], häufig Barrieren dar. So können fehlende Kontraste und die Verwendung von Fachtermini zur Hürde für Menschen mit Beeinträchtigung werden. Letztere erschweren oftmals den Zugang zu wesentlichen Informationen für Menschen mit Lernschwierigkeiten, welche aus vielerlei Gründen nur begrenzt im digitalen Raum teilhaben können [2]. Diese Personengruppe stellt den größten Anteil an Beschäftigten in Werkstätten für Menschen mit Behinderung (WfbM) dar [9]. Ein Blick in die Praxis der Werkstätten zeigt, dass es hier häufig an barrierearmen Konzepten und Materialien mangelt, die die Zielgruppe im Sinne der UN-BRK für Themen der Gesundheitsförderung und des Arbeitsschutzes informieren und befähigen. Besonders in dem Lebensbereich Arbeit, bei dem die Betriebe nach dem Arbeitschutzgesetz (ArbSchG) gesetzlich verpflichtet sind, die Beschäftigten vor gesundheitlichen Schädigungen zu schützen, werden WfbM bei der konzeptionellen Umsetzung bisher außer Acht gelassen [4]. Um das Gesundheitsverhalten von Menschen mit Lernschwierigkeiten positiv zu beeinflussen, ist es daher von Nöten, die vielfältigen Barrieren abzubauen und neue, ganzheitliche Konzepte zu entwickeln. Diese könnten dazu beitragen, die bestehende Lücke an barrierearmen Gesundheitskonzepten zu schließen und die Teilhabe an Arbeit und letztlich auch an der Gesellschaft zu stärken [21].

Ein wesentlicher Faktor bei der Entwicklung von Konzepten zum Arbeitsschutz und zur Gesundheitsförderung ist die Einbeziehung der Adressierten im Rahmen eines partizipativen Vorgehens. Bei diesem sollten die Adressierten mit ihrem Gesundheitsverständnis und ihren Ressourcen aktiv in den Prozess eingebunden werden [19]. Eine Möglichkeit, Lerninhalte zu Gesundheit und Arbeitsschutz partizipativ zu gestalten und auch in Zeiten von Kontaktbeschränkungen zu trainieren, bietet barrierearme, digitale Lernsoftware. Diese sollte zur Schulung von Menschen mit Lernschwierigkeiten eingesetzt werden, da diese privat und beruflich am häufigsten von digitaler Exklusion betroffen sind [8]. Häufige Gründe für die erschwerten Teilhabemöglichkeiten sind fehlende Internetzugängen und hinderlichen Strukturen im Arbeits- und Wohnsetting [6]. Infolgedessen bieten sich für Menschen mit Lernschwierigkeiten weniger Möglichkeiten, auf digitale Gesundheitsinformationen zuzugreifen. Die Stärkung digitaler Kompetenzen durch die Nutzung digitaler Lernangebote wäre ein gewünschter Nebeneffekt.

Beispielsweise bietet die sam®-Software von secova die Möglichkeit, auf digitale Weise den verpflichtenden Unterweisungen zum Arbeitsschutz kontrolliert nachzukommen sowie diese zielgruppenorientiert und praxisbezogen durchzuführen. Dies ermöglicht eine Alternative zu analogen Schulungen, welche für die Adressierten häufig zu langwierig sowie zu theoriebasiert gestaltet sind. Digitale Tools bieten die Möglichkeit, Inhalte zum selbst gewählten Zeitpunkt zu lernen, zu sequenzieren und dadurch die Komplexität der Inhalte beizubehalten. In der sam®-Software können Lerninhalte in einzelnen Lerneinheiten mit Videos, Fotos und Piktogrammen gestaltet werden. Auf diese Weise können bspw. Kompetenzen zum Thema Erste Hilfe ausgerichtet auf das jeweilige Arbeitssetting, wie z. B. die maschinelle Fertigung, vermittelt werden. Des Weiteren zeichnet sich diese Software dadurch aus, dass sich die Inhalte nach dem individuellen Lernverhalten ausrichten lassen und Wiederholungen sowie Quizabfragen möglich sind. Die Inhalte können barrierearm gestaltet werden, sodass sie für Menschen unterschiedlicher Bedarfe auffindbar, zugänglich und nutzbar sind [12]. Auf diese Weise könnte deren Motivation gesteigert sowie deren Vigilanz verstärkt werden.

Um eine solche Lernsoftware erfolgreich in den Arbeitsalltag einzuführen, sind grundsätzlich technische und personelle Voraussetzungen wie die dringende Förderung digitaler Kompetenzen [6] zu beachten. Der vorliegende Artikel stellt forschungsbasierte Ansätze vor, wie digitale und barrierearme Lerninhalte partizipativ gestaltet und genutzt werden können.

## Methode

Grundlage dieses Artikels ist ein Forschungsprojekt, das im Zeitraum von Oktober 2019 bis Mai 2020 von Studierenden der TU Dortmund in Kooperation mit den WfbM der Recklinghäuser Werkstätten gGmbH durchgeführt wurde. Ziel war es, in partizipativ gestalteten [1, 20] qualitativen Befragungen Gesundheits- und Medienkompetenzen zu erfragen sowie jene Aspekte zu erheben, die für die Adressierten die Zugänglichkeit und Nutzbarkeit von digitalen Lerninhalten erhöhen. Außerdem sollten im Prozess Beispiele zur Gesundheitsförderung in der Software als Diskussionsgrundlage entwickelt werden.

Zentrale Merkmale des partizipativen Forschungsansatzes sind der Teilhabeaspekt, die Ausrichtung des Forschungsvorhabens auf die Anwendung und die Veränderung des Forschungsfeldes sowie die Befähigung der Koforschenden [20]. Die dem Forschungsprojekt zugrunde liegende Fragestellung lautet: Welche Gestaltungsaspekte können für die digitale Vermittlung von Inhalten zu Arbeitsschutz und Gesundheitsförderung für Menschen mit Lernschwierigkeiten auf Basis qualitativer Erhebungen abgeleitet werden?

Die Datenerhebung erfolgte in Form von drei Fokusgruppentreffen (FGT) je 60 min [16] im Zeitraum vom 13.02. bis 12.05.2020 in Räumlichkeiten der WfbM. Die Teilnehmenden der ersten beiden FGT setzten sich aus drei Mitarbeitenden^2^ und fünf Beschäftigten^3^ der kooperierenden WfbM zusammen. Aufgrund der COVID-19-Pandemie fand das dritte FGT mit vier Mitarbeitenden per Videokonferenz statt. Ablauf und Durchführung der drei FGT orientierten sich am Ablaufmodell von Gruppendiskussionen nach Mayring [17] und an den Regeln zur Durchführung von FGT nach Lamnek und Krell [16]. Moderiert wurden die FGT von zwei Studierenden des Forschungsprojekts. Das erste FGT behandelte das Thema Gesundheit und enthielt thematische Grundreize für den ersten allgemeinen Austausch. Im zweiten FGT erfolgte der Austausch direktiver. Hier wurden folgende Themen diskutiert: Faktoren, die die eigene Gesundheit (am Arbeitsplatz) begünstigen und gefährden; der Wissensstand bzgl. Erste Hilfe; (Zweck der) Nutzung von digitalen Medien und dabei auftretende Schwierigkeiten; bevorzugte persönliche Lernstrategien; Anforderungen und Ideen zur Gestaltung der digitalen Lerninhalte in der sam®-Software. Auf Basis der FGT 1 und FGT 2 entwickelte die Projektgruppe Beispiele für Lerninhalte in der Software sowie einen ersten Entwurf für die Handreichung. Im dritten FGT wurden die Beispiele sowie die Handreichung evaluiert. Die erhobenen Daten wurden mittels einer qualitativen Inhaltsanalyse [17] ausgewertet. Die Handreichung wurde im Anschluss daran angepasst und ergänzt.

## Ergebnisse

Im Folgenden werden ausgewählte Ergebnisse bzgl. der Handreichung vorgestellt. Die Handreichung bietet Empfehlungen zur sprachlichen, inhaltlichen und graphischen Gestaltung sowie Leitlinien bzgl. didaktischer Methoden, die bei der Wissensvermittlung für die Adressierten mit Lernschwierigkeiten zu beachten sind. Zudem weist die Handreichung auf technische und rechtliche Voraussetzungen hin. Hilfreiche Links bieten tiefergehende Informationen zu ausgewählten Themen. Die Handreichung soll die Verantwortlichen in den Einrichtungen dabei unterstützen, die digitalen Lerninhalte, wie bspw. Informationen zur Arbeitskleidung, in der jeweiligen Einrichtung entsprechend der Bedarfe und Ressourcen der Adressierten barrierearm anzupassen.

Aus den Ergebnissen der empirischen Erhebung wird deutlich, dass der Aufbau der digitalen Lerneinheiten intuitiv nachvollziehbar sein sollte. So können Arbeitsaufforderungen wie folgt formuliert werden: „Hier können Sie ein Quiz machen. In dem Quiz geht es um das Thema *Erste Hilfe*. Im Quiz gibt es 3 Fragen. Das Quiz dauert etwa 10 min.“. Bei der sprachlichen Gestaltung sollten u. a. folgende Aspekte beachtet werden:

- Formulierung kurzer Sätze mit je einer Aussage,
- Vermeidung von:
  - komplexen Satzkonstruktionen,
  - Verneinungen,
  - Fragen,
  - Fach- oder Fremdwörtern.

Auf Basis der FGT werden für die graphische Gestaltung folgende mediale Formate für Lerninhalte empfohlen:

- Piktogramme,
- Bilder,
- Animationen,
- Videos,
- Texte in einfacher Sprache unterstützt mit Bildern und Piktogrammen.

Diese Vielfalt ermöglicht den Adressierten, das Medium ihrer Wahl nach eigenen Bedarfen und Vorlieben zu nutzen. Durch diese Vielfalt soll das Interesse und die Aufmerksamkeit erhalten werden. Personen mit einer eingeschränkten Lesefähigkeit sollten bspw. auf Erklärvideos und Animationen zurückgreifen können. Die Inhalte der medialen Formate sind klar und barrierearm zu gestalten. Die barrierefreie Gestaltung meint u. a.:

- Verwendung einer serifenlosen Schrift,
- Nutzung von Untertiteln,
- Verwendung von Audiodeskriptionen und Alternativtexten,
- Berücksichtigung einer übersichtlichen, reizarmen sowie kontrastreichen Gestaltung.

Ablenkende sensorische Reize wie dekorative Animationen oder Hintergrundmusik können laut den Befragten zu einer Überforderung und Blockade des Lernprozesses führen. Bild- und Videoaufnahmen sollten das Umfeld der Adressierten abbilden, z. B. durch Fotos vom Feuerlöscher im jeweiligen Arbeitsraum oder anhand selbstgedrehter Videos am Arbeitsplatz. Auf diese Weise können der Wissenstransfer, ein höheres Bewusstsein und die Aufmerksamkeit für die Lerninhalte verstärkt werden.

In den FGT wurde eine Vielfalt an persönlichen Lernmethoden geäußert, u. a. das Lernen von Inhalten, die mit Symbolbildern bspw. aus bekannten Datenbanken zur Unterstützten Kommunikation untermalt sind oder das Lernen anhand eines Quiz. Die Individualität des Lernens und eine Angebotsvielfalt sind stets zu berücksichtigen. Des Weiteren sollten die thematischen Inhalte in einzelne Abschnitte aufbereitet werden, um ein Aneignen im eigenen Lerntempo zu ermöglichen. Die Auswahl der Inhalte und der Zeitpunkt des Lernens sollten nach den Ressourcen und Bedarfen der Adressierten sowie den Kapazitäten vor Ort angepasst werden. In den Einrichtungen kann die Lernsituation als Gruppenarbeit, Einzelsetting oder als 1:1-Situation mit Mitarbeitenden gestaltet werden. Um das neu erworbene Wissen zu festigen, bietet sich die automatische Quizfunktion der Software mit verschiedenen Einstellungsmöglichkeiten und Text- sowie Bildinhalten an. Bei der Gestaltung der Kontrollfragen ist auf einen Schwierigkeitsgrad zu achten, der an die Adressierten angepasst ist. Ein Transfer des neu erworbenen Wissens könnte bspw. in Gruppensettings stattfinden.

## Diskussion

Das vorliegende Forschungsprojekt folgte anhand der heterogenen Fokusgruppe einem partizipativen Ansatz, indem durch einen offenen Interessenaustausch die Ideen und Meinungen der Beteiligten gesammelt und in den Entscheidungsprozess einbezogen wurden. Um die Chancengleichheit in den FGT zu stärken, wurde die Diskussion moderiert sowie eine einheitliche Ansprache aller Teilnehmenden gewählt. Zur Unterstützung der Kommunikation wurden Karten mit Symbolbildern eingesetzt und Ergebnisse in Bild und Schrift visualisiert. Insgesamt konnten Mitarbeitende und Beschäftigte aktiv in den Forschungsprozess eingebunden werden. Außerdem förderte der offene Gesprächscharakter der FGT die Thematisierung von weniger präsenten Inhalten und frei gewählten Themen der Beschäftigten, die für die Ausarbeitung der Gestaltungsmethoden hilfreich waren. Die vorangestellte Methodik bietet somit eine gute Handlungsrichtlinie, um einen offenen Kontakt aufzubauen und durch einen stetigen Austausch zielgruppengerechte Gestaltungsmethoden für die digitale Gesundheitsförderung zu entwickeln.

Es ist kritisch hervorzuheben, dass die Beschäftigten aufgrund der Maßnahmen zur Eindämmung der COVID-19-Pandemie und des Mangels an digitalen Zugängen in ihrem jeweiligen Wohnsetting nicht am letzten FGT teilnehmen konnten. So wurden die entwickelten Lerninhalte und die Handreichung lediglich durch die Mitarbeitenden evaluiert, wodurch eine mögliche Verzerrung bzgl. der Einschätzungen von Zugänglichkeit und Angemessenheit zu berücksichtigen ist. Um die Beschäftigten zukünftig stärker einbeziehen zu können, zeigt sich besonders im Hinblick auf die COVID-19-Pandemie die Notwendigkeit, technische Voraussetzungen zu schaffen, die die digitale Teilhabe von Menschen mit Lernschwierigkeiten stärken und den Zugang zur (digitalen) Gesundheitsförderung ermöglichen. Der Ausbau der digitalen Teilhabe setzt dabei den Erwerb von Medienkompetenzen aller Beteiligten in den Einrichtungen voraus [6, 10, 18]. Für die Einführung neuer digitaler Hilfsmittel in sozialen Einrichtungen ist die Orientierung an forschungsbasierten Workshopkonzepten ratsam [7].

Das Forschungsprojekt zielte auf die Erstellung einer Handreichung für die barrierefreie Gestaltung von digitalen Lerninhalten ab. Hierfür konnten forschungsbasierte Erkenntnisse abgeleitet werden. Aufgrund möglicher Verzerrungen sowie des empfohlenen partizipativen Vorgehens sollten die Handreichung und darauf aufbauende Lerninhalte in der Praxis evaluiert werden. Der Einsatz der Handreichung ist auch in anderen Settings für die Gestaltung von digitalen Lerninhalten denkbar, bspw. um Abläufe und Routinen in besonderen Wohnformen zu trainieren oder Menschen mit Lernschwierigkeiten im Ausbildungsprozess zu begleiten [7]. Zukünftig sollen auf Basis der Handreichung digitale Lerninhalte die analogen Schulungen in WfbM ersetzen. Denn diese sind häufig universell und zeitintensiv gestaltet. Hingegen kann ein digitales Format in Verbindung mit einer didaktischen Vielfalt einen barrierefreien und selbstständigen Zugang zum Lernen ermöglichen. Außerdem können im digitalen Format die Lerninhalte an die spezifischen Ausstattungsmerkmale der Einrichtung wie bspw. Sicherheitsregeln vor Ort angepasst werden. All dies kann dazu beitragen, die Beschäftigten zu einem gesundheitsförderlichen Verhalten zu befähigen. Dies kann die Chancengleichheit bzgl. des Erwerbs von Gesundheitskompetenzen von Menschen mit Lernschwierigkeiten stärken.

Das Forschungsprojekt leistet somit einen Beitrag dazu, die Lücke an barrierearmen Gesundheitskonzepten zu schließen und die Teilhabe an Gesundheit von Menschen mit Lernschwierigkeiten auszuweiten. Es stellt einen weiteren Schritt in die Richtung dar, allen Menschen ein Höchstmaß an Gesundheit zu ermöglichen und die gesetzlichen Verpflichtungen der UN-BRK umzusetzen.

## Fazit für die Praxis

- Die Studie konnte einen Beitrag zum Abbau von Barrieren in der digitalen Vermittlung von Arbeitsschutzmaßnahmen in WfbM (Werkstatt für Menschen mit Behinderung) leisten. Dadurch kann der Zugang zu Gesundheitsinformationen sowie die Teilhabe von Personen mit Lernschwierigkeiten langfristig gestärkt werden.
- Durch die partizipative Forschungsmethode können die Bedarfe und Anregungen der Adressierten in den Prozess sowie die Umsetzung einfließen. Die aktive Mitbestimmung von Menschen mit Behinderung sowie deren Teilhabe am Forschungsprozess ist zu fokussieren.
- In zukünftigen Forschungsprojekten sollte die Teilnahme an den Fokusgruppentreffen (FGT) auf weitere Beschäftigte sowie Mitarbeitende der WfbM ausgeweitet werden. Dabei sind individuelle Kommunikationsbedarfe zu berücksichtigen.

## Footnotes

1 Gemeint sind Personen, welche Schwierigkeiten beim Erreichen von bestimmten Lernzielen aufweisen.

2 Personen, die sozialversicherungspflichtig in den Recklinghäuser Werkstätten gGmbH tätig sind.

3 Personen, die als Leistungsempfänger*innen nicht sozialversicherungspflichtig in den Recklinghäuser Werkstätten gGmbH tätig sind.
